# Inhibitory effect of ultrasonic stimulation on the voltage-dependent potassium currents in rat hippocampal CA1 neurons

**DOI:** 10.1186/s12868-018-0485-1

**Published:** 2019-01-05

**Authors:** Kun Cui, Shuai Zhang, Jinyao Sun, Xueying Zhang, Chong Ding, Guizhi Xu

**Affiliations:** 1 State Key Laboratory of Reliability and Intelligence of Electrical Equipment, Hebei University of Technology, No. 8 Hongrong Road, Hongqiao District, Tianjin, 300132 China; 2 Key Laboratory of Electromagnetic Field and Electrical Apparatus Reliability of Hebei Province, Hebei University of Technology, Tianjin, 300132 China

**Keywords:** Ultrasonic stimulation, Delayed rectifier potassium current, CA1 pyramidal neuron, Transient outward potassium current, Patch clamp

## Abstract

**Background:**

Transcranial ultrasonic stimulation is a novel noninvasive tool for neuromodulation, and has high spatial resolution and deep penetration. Although it can increase excitation of neurons, its effects on neuron are poorly understood. This study was to evaluate effect of ultrasonic stimulation (US) on neurons in vitro. In this paper, the effect of US on the excitability and voltage-dependent $K^{ + }$ currents of CA1 pyramidal neurons in the rat hippocampus was studied using patch clamp.

**Results:**

Our results suggest that US increased the spontaneous firing rate and inhibited transient outward potassium current ($\varvec{I}_{\varvec{A}}$) and delayed rectifier potassium current ($\varvec{I}_{\varvec{K}} )$. Furthermore, US altered the activation of $\varvec{I}_{\varvec{K}}$ channels, inactivation and recovery properties of $\varvec{I}_{\varvec{A}}$ channels. After US, the $\varvec{I}_{\varvec{K}}$ activation curves significantly moved to the negative voltage direction and increased its slope factor. Moreover, the data showed that US moved the inactivation curve of $\varvec{I}_{\varvec{A}}$ to the negative voltage and increased the slope factor. Besides, US delayed the recovery of $\varvec{I}_{\varvec{A}}$ channel.

**Conclusions:**

Our data indicate that US can increase excitation of neurons by inhibiting potassium currents. Different US decreased the voltage sensitivity of $\varvec{I}_{\varvec{K}}$ activation differentially. Moreover, the more time is needed for US to make the $\varvec{I}_{\varvec{A}}$ channels open again after inactivating. US may play a physiological role by inhibiting voltage-dependent potassium currents in neuromodulation. Our research can provide a theoretical basis for the future clinical application of ultrasound in neuromodulation.

## Background

Therapeutic brain stimulation is a vital part of brain function research. Although they have been shown to be effective in treating neurological diseases, most of the current methods of stimulating the brain have some limitations. For instance, as a popular tool of brain stimulation, transcranial magnetic stimulation can modulate cognitive tasks while it is limited by poor spatial resolution [1–3]. Transcranial direct current stimulation also does not reach specific areas of the deep brain [4]. Deep brain stimulation has precise targeting specificity whereas requires surgery and electrode implantation [5]. Focused ultrasound can stimulate specific areas of nerve tissue with a diameter of a few millimeters [6]. Transcranial ultrasound stimulation (TUS) requires no surgery and has high spatial resolution and deep penetration [7–10]. William J. Tyler et al. determined low intensity and low frequency ultrasound (LILFU) can exciting neurons and network activity remotely and noninvasively. Their results indicate that LILFU can activate voltage-dependent Na^+^ channels and Ca^2+^ channels to induce neuronal activity [11]. Nicolas Wattiez et al. demonstrated that the neuromodulation effect of TUS on conscious behavioral monkeys can be assessed by real-time recording of discharge activity in brain regions connected to the stimulated region [12]. Using extracellular electrophysiology, Hongsun Guo and Mark Hamilton II et al. used TUS and performed brain mapping studies in guinea pigs. And they found an indirect auditory mechanism [13]. Whether it is the central nervous system or tumor, ultrasound provides a novel and effective strategy for targeted therapy [14–17].

Ultrasound stimulation (US) refers to TUS in vitro. US can produce the intramembrane mechano-electrical effect without tissue damage. US involves mechano-electrical coupling. Such coupling is linked to changes of capacitance [18, 19]. Inducing cavitation of lipid bilayer membranes, ultrasound can produce a mechano-electrical effect leading to neuronal excitation. It primarily is attributed to currents changes caused by the change of capacitance. The negative pressure of the ultrasonic waves pulled the leaflets apart each other whereas the positive pressure pushed forward. The average membrane capacitance is influenced by the dynamic deformed leaflets. Besides, ultrasound can affect mechanically sensitive ion channels to conduct currents [20]. The previous reported results verified that the activity of neurons can be excited by ultrasound through activation of some channel, which indicated great potential in the ultrasound therapy in ion channels [20–22].

Ion channels are excellent targets for diagnosis and therapy [23]. Whether as a major cause or as a mediator in the pathogenesis, they are involved in many diseases, such as epilepsy. In membranes of excitable and inexcitable cells, potassium channels are common and abundant [24, 25]. By setting the membrane potential, potassium channels regulate the electrical excitability of the neurons, which is a major function of potassium channels. Furthermore, K^+^ channel activity exerts an enormous function on signal pathways, among cell proliferation, differentiation and fusion [26, 27]. It is necessary to promote calcium entry that increased *K*^+^ channel activity and enhanced potassium efflux maintenance membrane hyperpolarization [28]. Besides, additional pathways for potassium channels such as to control the cell volume, are thought to involve in cell proliferation for which the membrane hyperpolarization is an essential requirement [29, 30]. For shaping the action potential, voltage-dependent potassium currents are important and can be divided into rapidly inactivating currents $\varvec{I}_{\varvec{A}}$ and non-inactivating currents $\varvec{I}_{\varvec{K}}$ broadly [31, 32].

In our studies, we recorded the firing rates and the total current by whole-cell patch clamp apparatus and speculated that US affects potassium currents, which caused the increased spontaneous action potential frequency. Here, we investigated the impact of the US on potassium current, which is a major part of the outward current. During neuronal excitation, voltage-dependent potassium currents play a significant role in making the depolarized cell resting [33]. During the repolarizing phase, $\varvec{I}_{\varvec{A}}$ and $\varvec{I}_{\varvec{K}} \varvec{ }$ are the main currents of the neuronal action potential [34]. In this study, we observed whether US has effects on $\varvec{I}_{\varvec{A}}$ and $\varvec{I}_{K}$ of CA1 pyramidal neurons.

## Methods

### Brain slices preparation

1–2 weeks old juvenile Sprague–Dawley male rats, were purchased from Chinese People’s Liberation Army Academy of Military Medical Laboratory Animal Center. Upon arrival, the rats were placed in a 23 ± 1 °C chamber with a cycle of 12 h day and 12 h night. Veterinarians performed standard monitoring for a period of time prior to the experiment. Rats can get diet. The experimental animal studies were worked on the basis of institutional guidelines for animal experiments and the International Pain Research Association’s ethical guidelines. Brain slice cultures were prepared from the rats. The intraperitoneal of rats were injected with pentobarbital (130 mg/kg), and then the rats were anaesthetized. The anesthetized rats were decapitated, and the brains were removed. The brains were transferred into cold (0 °C) slicing solution (in mmol: 2.40 KCl, $6.00   {\text{MgCl}}_{ 2}$, 1.00 ${\text{CaCl}}_{ 2}$, 24.50 ${\text{NaHCO}}_{3}$, 1.25 ${\text{NaH}}_{2} {\text{PO}}_{4}$, 11.00 glucose, 225.00 sucrose; adjusting pH 7.4, with KOH and HCl) aerated with a mixture of gas with 95% ${\text{O}}_{2}$ and 5% ${\text{CO}}_{2}$ for 30 s. In the ice cold slicing solution, the brain was rapidly cut into hemispheres, and 330 μm thick horizontal slices was prepared by using a microtome (VT1200S, Leica, Nussloch, Germany). The brain tissue slices were cultured at a temperature of 37–39 °C inside a holding chamber on an interface between oxygenated artificial cerebrospinal fluid (aCSF; in mmol: 11.00 glucose, 3.00 KCl, 2.00 ${\text{CaCl}}_{ 2}$, 2.00 ${\text{MgCl}}_{ 2}$, 123.00 NaCl, 1.25 ${\text{NaH}}_{2} {\text{PO}}_{4}$, 24.50 ${\text{NaHCO}}_{3}$; adjusting pH 7.4 with KOH and HCl) and 95% ${\text{O}}_{2}$/5% ${\text{CO}}_{2}$ for at least 45 min.

### Stimulation protocol

**Fig. 1:**
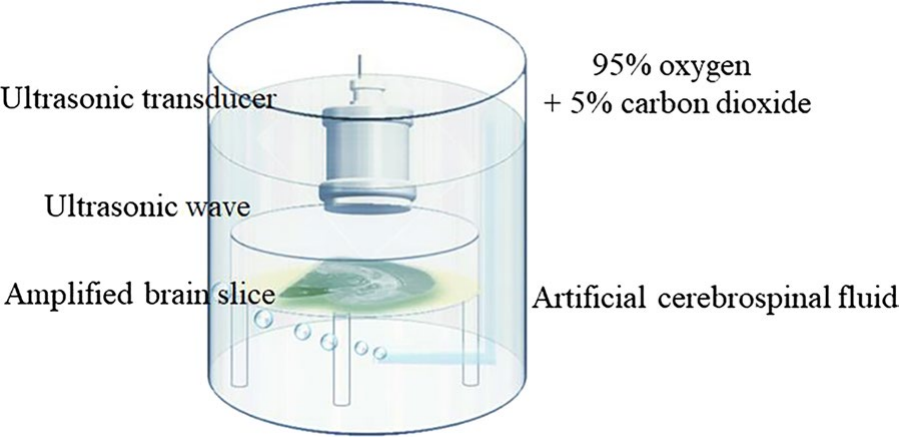
Ultrasonic stimulation of brain slice

The brain slice culture chamber is composed of a holder with a cylinder in a beaker and an outer wall. The pulsed ultrasound signals were generated by a radio-frequency power amplifier (Model 150A 100C, AR, WA, USA), an ultrasonic transducer (V308, Olympus, Tokyo, Japan) and an arbitrary waveform generator (33500B, KEYSIGHT, CA, USA). Ultrasonic transducer immersed in aCSF and was 1.5 cm above brain slice. The ultrasonic beam produced by the transducer (diameter = 24 mm) stimulates the entire brain slice (length < 9 mm, width < 6 mm). The ultrasound settings were 0.5 MHz center frequency, 20 Hz pulse repetition frequency, 50% duty cycle and 20 ms pulse length, and the pulse-average ultrasound intensities were 15 ${\text{mW}}/{\text{cm}}^{2}$ or 30 ${\text{mW}}/{\text{cm}}^{2}$. The current of brain slice without any stimulation and with ultrasonic stimulations were recorded as a control status (CTRL), 15 ${\text{mW}}/{\text{cm}}^{2}$ ultrasonic stimulation status (15 ${\text{mW}}/{\text{cm}}^{2}$ US) and 30 ${\text{mW}}/{\text{cm}}^{2}$ ultrasonic stimulation status (30 ${\text{mW}}/{\text{cm}}^{2}$ US, Fig. 1), respectively. The number of rats for each control and experimental group is 12 (n = 12). Four brain slices of each rat were used for control and experimental groups. All stimulations lasted 15 min.

### Patch clamp electrophysiology

Bathing in aCSF, the slices were visualized using infrared differential interference contrast microscopy (U-TV1X-2, Olympus, Tokyo, Japan). The voltages and currents of neuron cell membrane were collected by the amplifier of the patch clamp (EPC 10, HEKA, Pfalz, Germany). Borosilicate glass pipettes with resistances ranging from 4 to 9 MΩ. The pipettes were shaped by using the laser micropipette puller (MODEL P97, Sutter Instruments, CA, USA). All experiments were at 24 °C. The data was sampled at a frequency of 20 kHz, and was filtered at a frequency of 2 kHz using Patchmaster software. For recording of spontaneous action potential, pipettes were filled with the solution (in mmol): 133.00 K-gluconate, 2.00 MgCl_2_, 2.00 MgATP, 10.00 EGTA, 10.00 HEPES (keeping pH 7.4 with KOH and HCl). When the action potential was recorded, brain culture was in aCSF. For the recording of potassium currents, we filled the pipettes with the solution (in mmol): 2.00 CaCl_2_, 121.00 KCl, 10.00 EGTA, 1.00 MgCl_2_, 10.00 HEPES, 3.00 Na_2_ATP (keeping pH 7.4, with KOH and HCl). When the patch clamp experiments were performed, brain culture was bathed in a recording solution (in mmol) 6.00 KCl, 1.00 MgCl_2_, 130.00 NaCl, 2.00CaCl_2_, 10.00 Glucose, 10.00 HEPES (pH 7.4 with KOH and HCl). When transient outward potassium current was indicated, it was pharmacologically isolated with (in mmol) 20.00 TEA-Cl, 0.10 CdCl_2_ and 0.001 Tetrodotoxin (TTX) injected to the bath solution to block the other channels. When delay rectifier potassium current was indicated, it was pharmacologically isolated with (in mmol) 4.00 AP, 0.10 CdCl_2_ and 0.001 TTX injected to the bath solution. Three hippocampal CA1 neurons were recorded on each brain slice from 12 different rats respectively.

### Statistical analysis

Statistical analysis was performed using Patchmaster (HEKA, Pfalz, Germany), Origin Pro 8.0 (OriginLab, Hampton, VA, USA), GraphPad Prism 7.0 (GraphPad Software, CA, USA) and SPSS 23 (IBM, NY, USA). The recording data were calculated as mean ± SEM. We used one-way analysis of variance to statistically analyze the data. When *P* < 0.05, the data were considered significant.

## Results

**Fig. 2:**
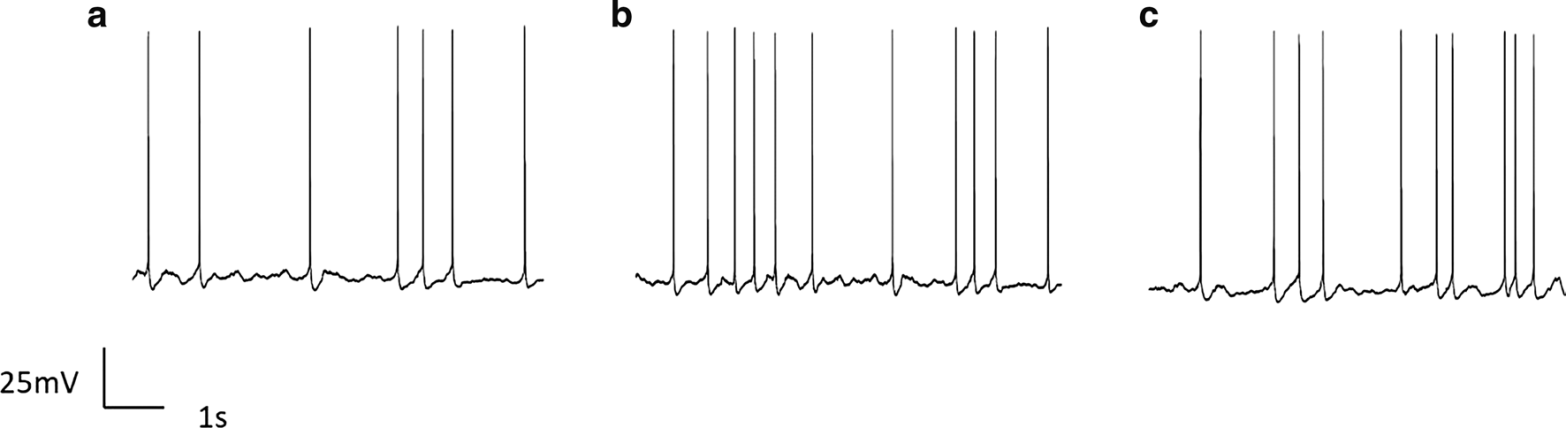
Effects of US on spontaneous firing action potentials of neurons. **a** Control. **b** 15 mW/cm^2^ US. **c** 30 mW/cm^2^ US

The spontaneous action potentials were recorded without current injection for 6 s in the current-clamp mode (Fig. 2). Both the action potential frequency and amplitude in neurons were analyzed (Table 1). There were significant the increased firing frequency and amplitude of action potentials both in 15 ${\text{mW}}/{\text{cm}}^{2}$ US and 30 ${\text{mW}}/{\text{cm}}^{2}$ US. It indicated that the exposure to US increased excitability of the neurons.

**Table 1:** Effects of stimulations on spontaneous action potentials of neurons

Group	Frequency	Amplitude
Control	1.19 ± 0.32	101.91 ± 4.19
15 mW/cm^2^ US	1.80 ± 0.29*	106.17 ± 3.29*
30 mW/cm^2^ US	1.65 ± 0.27*	105.33 ± 3.72*

n = 12, mean ± SEM

*P < 0.05 versus control

**Fig. 3:**
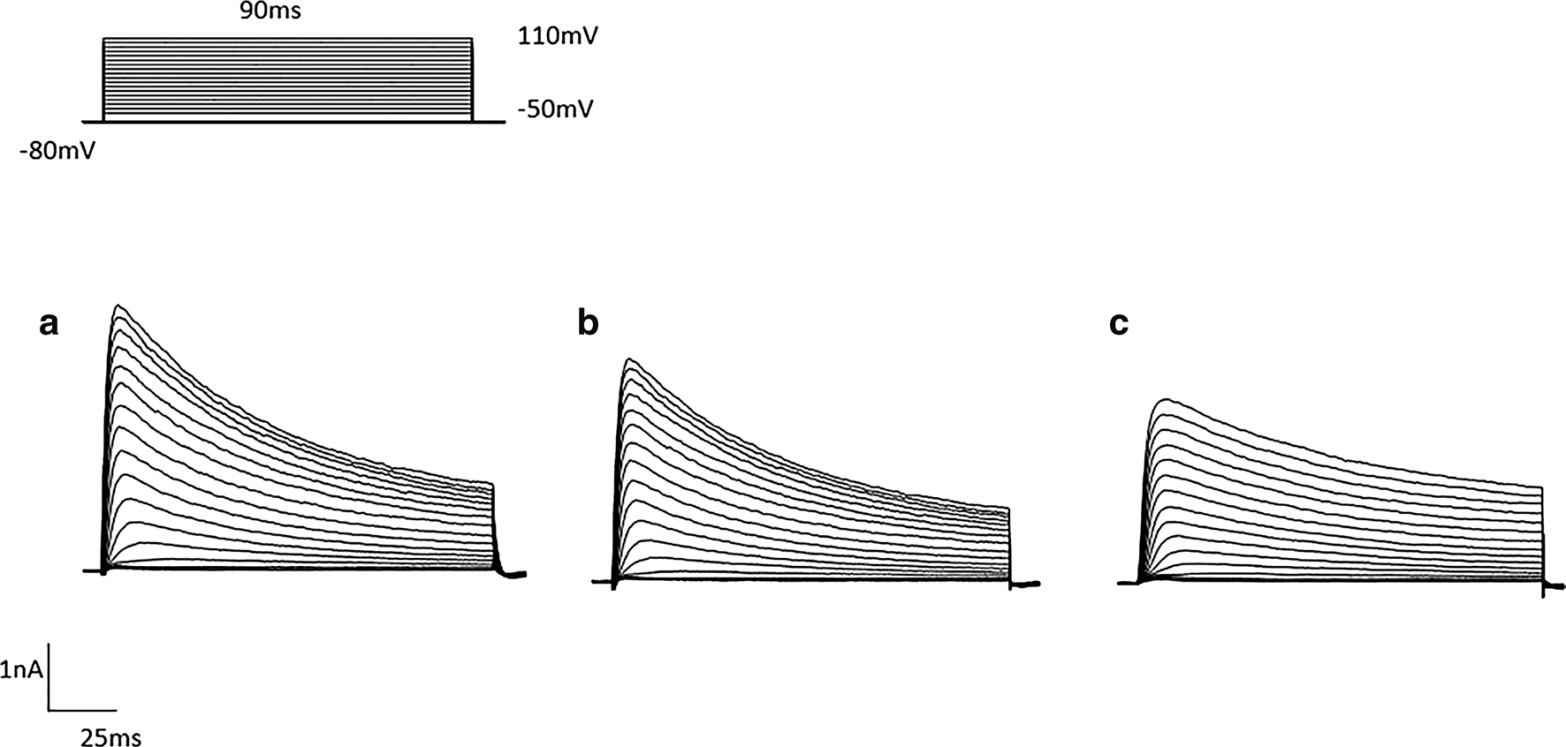
***I***_***A***_ of neurons after different stimulation. **a** Control. **b** 15 mW/cm^2^ US. **c** 30 mW/cm^2^ US

**Fig. 4:**
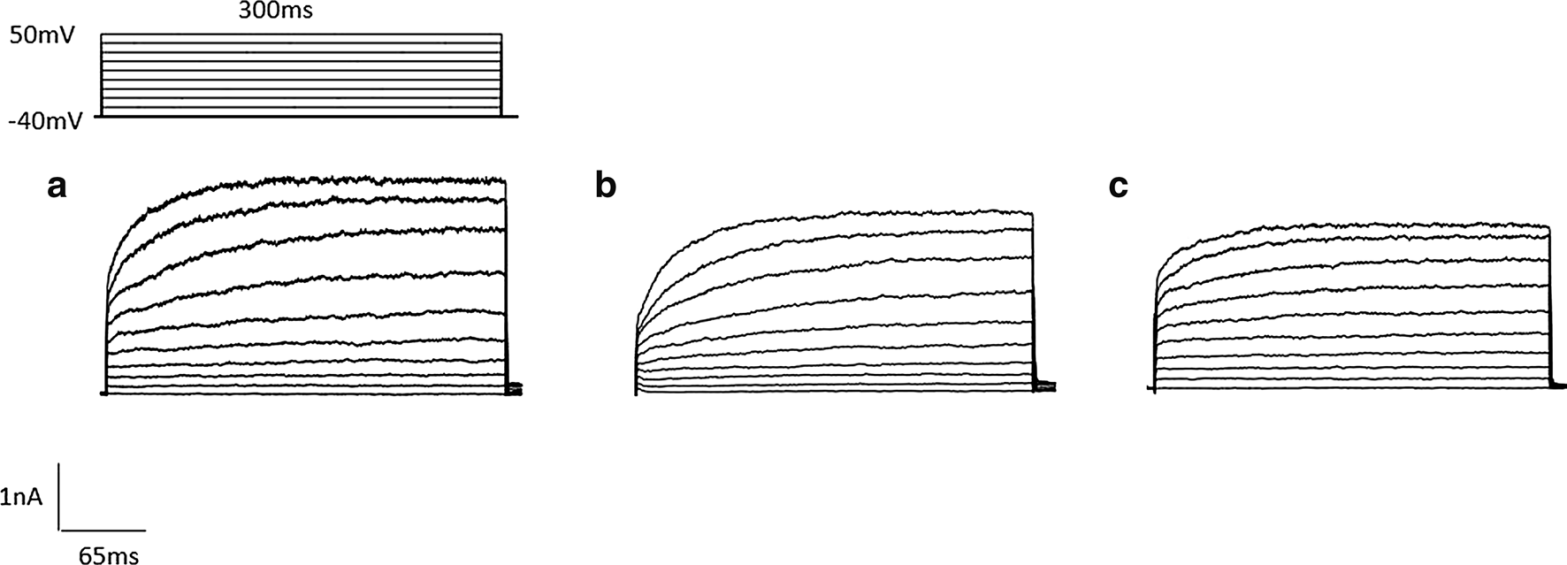
*I*_*K*_ of neurons after different stimulation. **a** Control. **b** 15 mW/cm^2^ US. **c** 30 mW/cm^2^ US

**Fig. 5:**
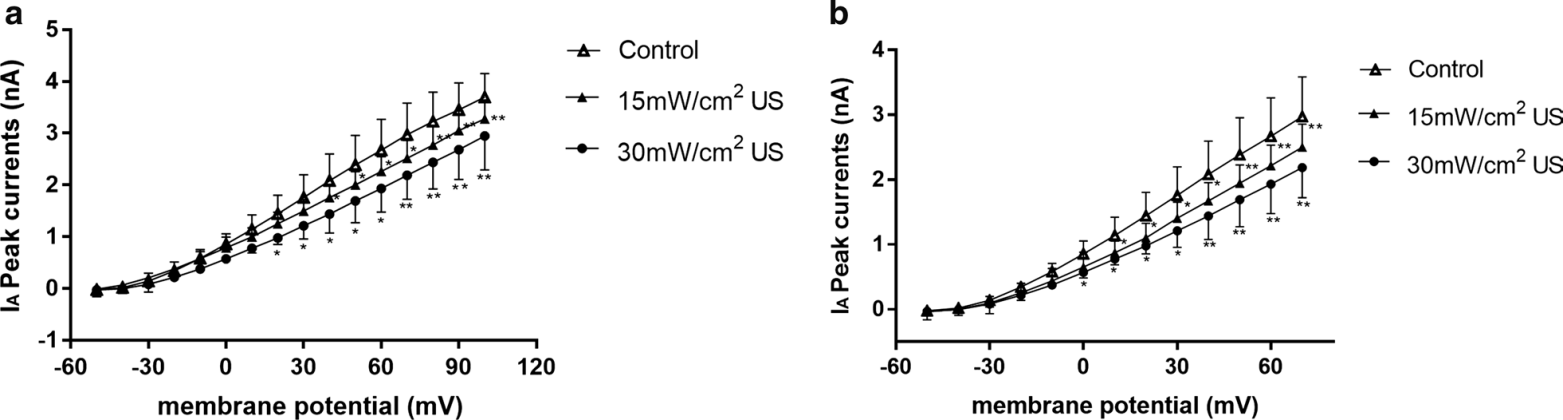
The current–voltage relationship of **a** ***I***_***A***_ and **b** ***I***_***K***_. Data are represented as mean ± SEM (n = 12, *P < 0.05 vs. control, **P < 0.01 vs. control)

For the recording of potassium currents, the membrane was maintained at a voltage of − 80 mV, and a 90 ms voltage pulse from − 50 to + 100 mV was applied in increase of 10 mV. When delay rectifier potassium currents were indicated, the membrane was at a voltage of − 40 mV and 300 ms voltage pulses were applied from − 40 to + 50 mV in incremental steps of 10 mV. The application of different US to brain slices produced obvious effects on the amplitudes of $\varvec{I}_{\varvec{A}}$ and $\varvec{I}_{\varvec{K}}$ in a different way (Figs. 3 and 4). For neurons exposed to 15 ${\text{mW}}/{\text{cm}}^{2}$ US and 30 ${\text{mW}}/{\text{cm}}^{2}$ US, the amplitudes of $\varvec{I}_{\varvec{A}}$ and $\varvec{I}_{\varvec{K}}$ were significantly lower than the Control neurons, which were indicated by current–voltage curves (Fig. 5). Therefore, we could determine that the potassium current values of US exposed neurons were significantly lower than other neurons.

**Fig. 6:**
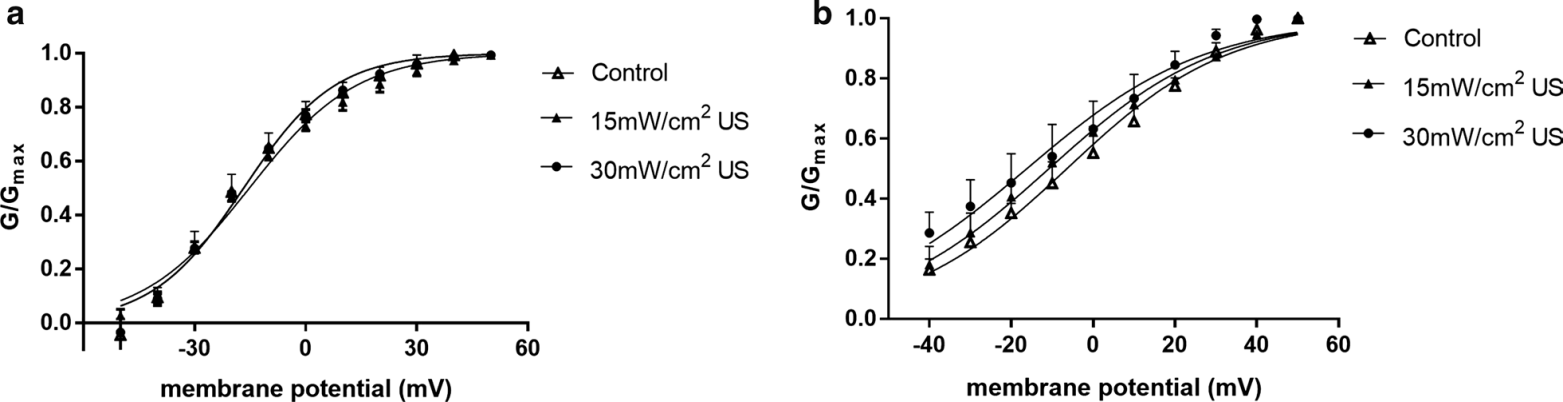
The steady-state activation curves of **a** ***I***_***A***_ and **b** ***I***_***K***_ after different stimulation

The conductance was calculated by $\varvec{G} = \varvec{I}/\left( {\varvec{V}_{\varvec{m}} - \varvec{V}_{{\varvec{rev}}} } \right)$. $\varvec{I}$, $\varvec{V}_{\varvec{m}}$ and $\varvec{V}_{{\varvec{rev}}}$ denotes current density, the membrane voltage and the channel reversal potential. Figure 6a, b showed activation curves of $\varvec{I}_{\varvec{A}}$ and $\varvec{I}_{\varvec{K}}$ after different stimulations respectively. We fitted the curves with a Boltzmann equation: $\varvec{G}/\varvec{G}_{{\varvec{max}}} = \varvec{I}/\left\{ {1 + \varvec{exp}\left[ {\left( {\varvec{V}_{\varvec{m}} - \varvec{V}_{\varvec{h}} } \right)/\varvec{k}} \right]} \right\}$, in which $\varvec{V}_{\varvec{h}}$ was the potential value in the semi-active state, $k$ was the slope factor. The effect of stimulation on $\varvec{I}_{\varvec{A}} \varvec{ }$ and $\varvec{I}_{\varvec{K}}$ activation parameters was summarized in Table 2. It indicated that 15 ${\text{mW}}/{\text{cm}}^{2}$ US and 30 ${\text{mW}}/{\text{cm}}^{2 }$ US have no significant effect on the activation characteristics of $\varvec{I}_{\varvec{A}}$. Furthermore, 15 ${\text{mW}}/{\text{cm}}^{2}$ US and 30 ${\text{mW}}/{\text{cm}}^{2}$ US induced a negative movement in this curve and decrease the slope factor.

**Table 2:** Effects of stimulations on the activation parameters of ${\text{I}}_{\text{A}}$ and ${\text{I}}_{\text{K}}$

Groups	***I***_***A***_		***I***_***K***_	
	*V*_*h*_	k	*V*_*h*_	k
Control	− 16.76 ± 2.32	12.45 ± 2.03	− 6.45 ± 2.61	19.87 ± 2.49
15 mW/cm^2^ US	− 15.19 ± 1.72	14.54 ± 1.57	− 10.83 ± 1.83*	20.60 ± 1.83
30 mW/cm^2^ US	− 16.68 ± 1.91	12.40 ± 1.68	− 16.38 ± 4.09*	22.20 ± 4.05*

n = 12, mean ± SEM

*P < 0.05 versus control

**Fig. 7:**
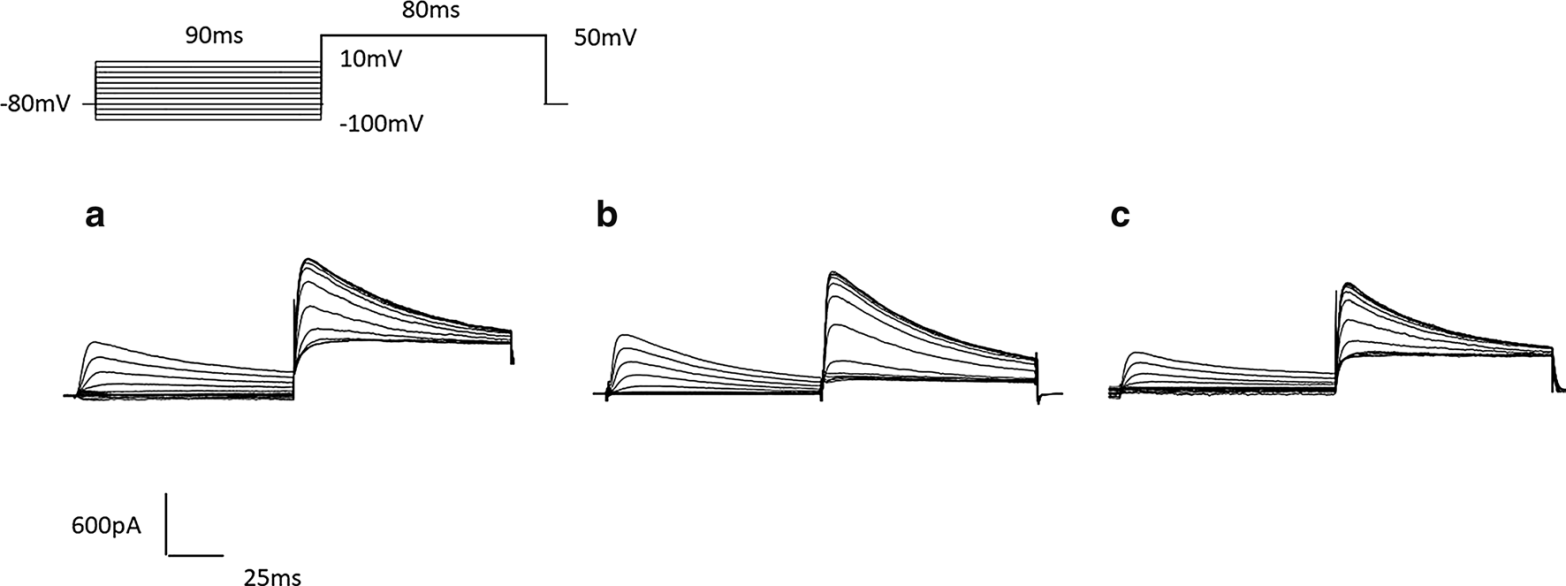
The inactivation of ***I***_***A***_ after different stimulation. **a** Control. **b** 15 mW/cm^2^ US. **c** 30 mW/cm^2^ US (n = 12, mean ± SEM)

The inactivation characteristics of $\varvec{I}_{\varvec{A}} \varvec{ }$ were recorded by the double-pulse protocols (Fig. 7). But the inactivation of $\varvec{I}_{\varvec{K}}$ was not recorded because it is a type of long-lasting channels. The membrane was maintained at − 80 mV, changed to varying 90 ms prepulse voltages from − 100 to 10 mV in increase of 10 mV and to an 80 ms test pulse at + 50 mV. After the peak amplitude of $\varvec{I}_{\varvec{A}}$ was normalized, it was drawn with above prepulse potential. We fitted the inactivation curves of $\varvec{I}_{\varvec{A}}$ with Boltzmann equation $\varvec{I}/\varvec{I}_{{\varvec{max}}} = \varvec{I}/\left\{ {1 + \varvec{exp}\left[ {\left( {\varvec{V}_{\varvec{h}} - \varvec{V}_{\varvec{m}} } \right)/\varvec{k}} \right]} \right\}$, there $\varvec{I}/\varvec{I}_{{\varvec{max}}}$ was a normalized current of $\varvec{I}_{\varvec{A}}$, $\varvec{V}_{\varvec{h}}$ was the semi-inactivation voltage value, k was the curve’s slope factor (Fig. 9a). Both 15 ${\text{mW}}/{\text{cm}}^{2}$ and 30 ${\text{mW}}/{\text{cm}}^{2}$ US could significantly shift inactivation curve of $\varvec{I}_{\varvec{A}}$ to negative voltage direction and enhance its slope factor (Table 3).

**Table 3:** Effects of stimulations on the inactivation parameters of ***I***_***A***_

Groups	***I***_***A***_	
	*V*_*h*_	k
Control	− 39.26 ± 0.85	7.72 ± 0.78
15 mW/cm^2^ US	− 45.33 ± 2.32*	9.05 ± 2.08*
30 mW/cm^2^ US	− 44.31 ± 2.86*	8.62 ± 2.55*

n = 12, mean ± SEM

*P < 0.05 versus control

**Fig. 8:**
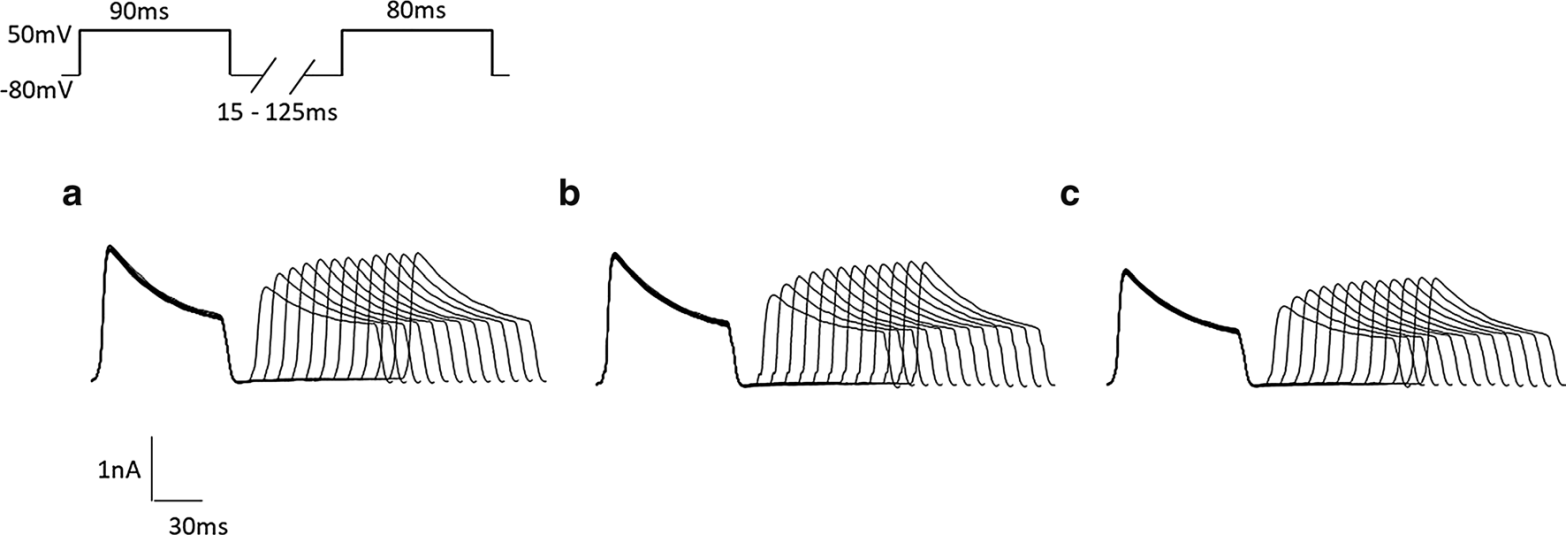
The recovery from inactivation of ${\text{I}}_{\text{A}}$ after different stimulation. **a** Control. **b** 15 mW/cm^2^US. **c** 30 mW/cm^2^US

In order to comprehend the recovery properties from inactivation, the membrane was maintained at − 80 mV and stepped up to + 50 mV for 90 ms (depolarizing pulse), then repolarized to − 80 mV varying from 15 to 125 ms in 10 ms increments before a test pulse of + 50 mV for 90 ms (Fig. 8). The amplitude of $\varvec{I}_{\varvec{A}}$, caused by above conditioning pulse, was defined as ***I***_1_, and ***I***_2_ was the peak current amplitude of the $\varvec{I}_{\varvec{A}}$ induced by test pulse. The characteristics of recovery after the inactivation can be analyzed using the value of ***I***_2_/***I***_1_. We fitted the ***I***_2_/***I***_1_ time curve with a mono-exponential equation:

$$ I/I_{\hbox{max} } = A + B\exp ( - t/\tau ). $$

**Fig. 9:**
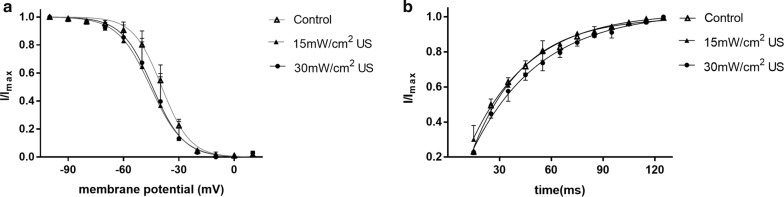
**a** The inactivation curves of ${\text{I}}_{\text{A}}$. **b** recovery after inactivation curves of ${\text{I}}_{\text{A}}$

***I*** is ***I***_2_/***I***_1_ and ***I***_max_ is the maximal value of ***I*** and, *τ* is the time constant (Fig. 9b). Table 4 showed the time constants *τ*. The results indicated that 15 mW/cm^2^ US and 30 mW/cm^2^ US could markedly increase the time constant of the recovery. Besides, 15 mW/cm^2^ US and 30 mW/cm^2^ US shifted the recovery from inactivation curve of ***I***_*A*_ to negative potential.

**Table 4:** Effects of stimulations on the recovery from inactivation parameters of ***I***_***A***_

Group	*τ*
Control	30.58 ± 4.59
15 mW/cm^2^ US	35.46 ± 6.09*
30 mW/cm^2^ US	40.25 ± 6.39*

n = 12, mean ± SEM

*P < 0.05 versus control

## Discussion

There are many evidences supporting the fact that US has neuronal effects [16, 22]. However, the lasting effects of US on neuronal excitability are not entirely clarified, especially the ion channel mechanism. Our study demonstrates that US can increase the frequency, amplitude the duration of spontaneous action potential to enhance excitation of neurons. As a result of US, the durations of single action potential were prolonged. Therefore it might be inferred that the increasing frequency would be responsible for the delay of repolarization which depends on $\varvec{I}_{\varvec{A}}$ and $\varvec{I}_{\varvec{K}}$.

Participating in early polarization, $\varvec{I}_{\varvec{A}}$ is crucial to the spike threshold. $\varvec{I}_{\varvec{K}}$ cause the repolarization. The width of spike also depends on $\varvec{I}_{\varvec{K}}$ [32, 35]. In addition, $\varvec{I}_{\varvec{K}}$ is critical for post-peak hyperpolarization and affects the peak frequency of neurons.

Therefore, to identify the ion channel mechanisms, we examined ion channels by analyzing the changes of $K^{ + }$ currents characteristics by patch clamp recording. The results revealed that US enhanced excitability of neurons in CA1 pyramidal neurons of rat hippocampal, which may be mediated by a reduction of potassium currents. The US effectively inhibited $\varvec{I}_{\varvec{A}}$ and $\varvec{I}_{\varvec{K}}$, and this effects of 30 ${\text{mW}}/{\text{cm}}^{2}$ US were more than 15 ${\text{mW}}/{\text{cm}}^{2}$ US.

Furthermore, US significantly moved the activation curves of $\varvec{I}_{\varvec{K}}$ to the negative voltage. It is shown that different US affected the activation of $\varvec{I}_{\varvec{K}}$ differentially. Besides, US increased the slope factor for $\varvec{I}_{\varvec{K}}$ activation curve, indicating that the voltage sensitivity of activation reduced. Moreover, the data showed that US moved inactivation curve of $\varvec{I}_{\varvec{A}}$ to the negative voltage and increased its slope factor. Besides, US delayed the recovery of $\varvec{I}_{\varvec{A}}$. This means that the $\varvec{I}_{\varvec{A}}$ channel takes longer to open again after inactivation. These results suggest that US inhibited $\varvec{I}_{\varvec{A}}$ and $\varvec{I}_{\varvec{K}}$ via reducing the open number of $\varvec{I}_{\varvec{A}}$ and $\varvec{I}_{\varvec{K}}$ channels.

## Conclusions

US can enhance neural excitation to activate the brain area, thereby altering the physiological processes in the brain. Potassium currents made the depolarized cell rest and are important during the action potential repolarizing. US can inhibit both $\varvec{I}_{\varvec{A}}$ and $\varvec{I}_{\varvec{K}}$ to increase excitation of neurons, particularly in high intensity of US. In this sense, the fact that US enhance excitation of neuron and act differently on potassium currents could potentially be used to design neuromodulation tools for neurological diseases. Besides, ultrasound can provide a method for targeted ion channels therapy almost no side effects. In clinical use, ultrasound is a promising treatment for the diseases to improving excitability in certain brain area. For example, ultrasound may be achieve the purpose of treating neurological diseases such as treatment of dyskinesia, epilepsy, stroke sequelae by changing the local cortical excitability of the brain. This study provide a theoretical basis for clinical ultrasound application in neuromodulation.

## Abbreviations

TUS: transcranial ultrasonic stimulation
US: ultrasonic stimulation
LILFU: low-intensity, low-frequency ultrasound
$\varvec{I}_{\varvec{A}}$: transient outward potassium current tetrodotoxin
TTX: tetrodotoxin
$\varvec{I}_{\varvec{K}}$: delayed rectifier potassium currents
aCSF: artificial cerebrospinal fluid
